# Global biochemical and structural analysis of the type IV pilus from the Gram-positive bacterium *Streptococcus sanguinis*

**DOI:** 10.1074/jbc.RA118.006917

**Published:** 2019-03-05

**Authors:** Jamie-Lee Berry, Ishwori Gurung, Jan Haug Anonsen, Ingrid Spielman, Elliot Harper, Alexander M. J. Hall, Vivianne J. Goosens, Claire Raynaud, Michael Koomey, Nicolas Biais, Steve Matthews, Vladimir Pelicic

**Affiliations:** From the ‡ Medical Research Council Centre for Molecular Bacteriology and Infection, Imperial College London, London SW7 2AZ, United Kingdom,; the § Department of Biological Sciences, Proteomics and Mass Spectrometry Unit, University of Oslo, 0371 Oslo, Norway,; the ¶ Department of Biological Sciences, Center for Integrative Microbial Evolution, University of Oslo, 0371 Oslo, Norway,; the ‖ Department of Biology, Brooklyn College of the City University of New York, New York, New York 11210,; ** The Graduate Center of the City University of New York, New York, New York 10016, and; the ‡‡ Centre for Structural Biology, Imperial College London, London SW7 2AZ, United Kingdom

**Keywords:** Gram-positive bacteria, protein structure, type IV pili, virulence factor, molecular motor, pilin, Streptococcus sanguinis, twitching motility, type IV filaments

## Abstract

Type IV pili (Tfp) are functionally versatile filaments, widespread in prokaryotes, that belong to a large class of filamentous nanomachines known as type IV filaments (Tff). Although Tfp have been extensively studied in several Gram-negative pathogens where they function as key virulence factors, many aspects of their biology remain poorly understood. Here, we performed a global biochemical and structural analysis of Tfp in a recently emerged Gram-positive model, *Streptococcus sanguinis*. In particular, we focused on the five pilins and pilin-like proteins involved in Tfp biology in *S. sanguinis*. We found that the two major pilins, PilE1 and PilE2, (i) follow widely conserved principles for processing by the prepilin peptidase PilD and for assembly into filaments; (ii) display only one of the post-translational modifications frequently found in pilins, *i.e.* a methylated N terminus; (iii) are found in the same heteropolymeric filaments; and (iv) are not functionally equivalent. The 3D structure of PilE1, solved by NMR, revealed a classical pilin-fold with a highly unusual flexible C terminus. Intriguingly, PilE1 more closely resembles pseudopilins forming shorter Tff than *bona fide* Tfp-forming major pilins, underlining the evolutionary relatedness among different Tff. Finally, we show that *S. sanguinis* Tfp contain a low abundance of three additional proteins processed by PilD, the minor pilins PilA, PilB, and PilC. These findings provide the first global biochemical and structural picture of a Gram-positive Tfp and have fundamental implications for our understanding of a widespread class of filamentous nanomachines.

## Introduction

Type IV pili (Tfp)^4^ are thin, long and flexible surface-exposed filaments, widespread in Bacteria and Archaea, which mediate a wide array of functions (1, 2). Tfp are polymers of primarily one major protein subunit, a type IV pilin with distinctive N-terminal sequence motif (class III signal peptide) and 3D structure (3), assembled by a conserved set of dedicated proteins (4). These defining features are shared by a large class of filamentous nanomachines called type IV filaments (Tff) (1). Tff are ubiquitous in prokaryotes because genes encoding type IV pilins and filament assembly proteins are found in virtually every bacterial and archaeal genome (1).

Much of our current understanding of Tff biology comes from studies of bacterial Tfp in a few Gram-negative human pathogens in which they function as key virulence factors (4). The following general picture has emerged. Prepilins are translocated by the general secretory pathway across the cytoplasmic membrane (5, 6), where they remain embedded via a universally conserved structural feature, *i.e.* a protruding hydrophobic N-terminal α-helix (α1N) (3). This leaves the hydrophylic leader peptide of the class III signal peptide in the cytoplasm, which is then cleaved by a membrane-bound aspartyl protease (7), the prepilin peptidase, generating a pool of mature pilins in the membrane ready for polymerization into filaments. Efficient prepilin processing by the prepilin peptidase, which does not require any other protein (8), depends on the last residue of the prepilin leader peptide (a conserved Gly) (9) and two conserved catalytic Asp residues in the prepilin peptidase (7, 10). How filaments are polymerized remains poorly understood, but it is clear that this process is mediated by a multiprotein machinery in the cytoplasmic membrane (11, 12), which transmits energy generated by a cytoplasmic hexameric assembly ATPase (13) to membrane-localized pilins. As a result, pilins are extruded from the membrane and polymerized into helical filaments via hydrophobic packing of their α1N helix within the filament core (14, 15). Finally, once Tfp reach the outer membrane, they are extruded onto the surface through a multimeric pore, the secretin (12, 16, 17). The above picture, although complex, is oversimplified because there are additional proteins that play key roles in Tfp biology, including several proteins with class III signal peptides, named minor pilins or pilin-like proteins, whose localization and exact role are often unclear. Moreover, Tfp are highly dynamic filaments, constantly extending and retracting. Retraction has been best characterized in a subclass known as Tfpa, where it results from filament depolymerization powered by the cytoplasmic hexameric ATPase PilT (18), which generates massive tensile forces (19, 20).

Until recently, Tfp have not been extensively studied in Gram-positive species although it has been recognized that this represents a promising new research avenue as these bacteria possess a simpler surface architecture (21). Although Gram-positive Tfp were first described in Clostridiales (22, 23), *Streptococcus sanguinis* has emerged as a model because it is genetically tractable (24, 25). A comprehensive genetic analysis of *S. sanguinis* Tfpa (24) has revealed that they: (i) are assembled by a similar machinery as in Gram-negative species but with fewer components, (ii) are retracted by a PilT-dependent mechanism, generating tensile forces very similar to those measured in Gram-negative species, and (iii) power intense twitching motility. The main peculiarity of *S. sanguinis* filaments is that they contain two major pilins, rather than one as normally seen (24). In the present study, we have focused on the pilins and pilin-like proteins involved in Tfp biology in *S. sanguinis* and have performed a global biochemical and structural analysis of its filaments.

## Results

### The pil locus in S. sanguinis 2908 encodes five pilin/pilin-like proteins

**Figure 1.**
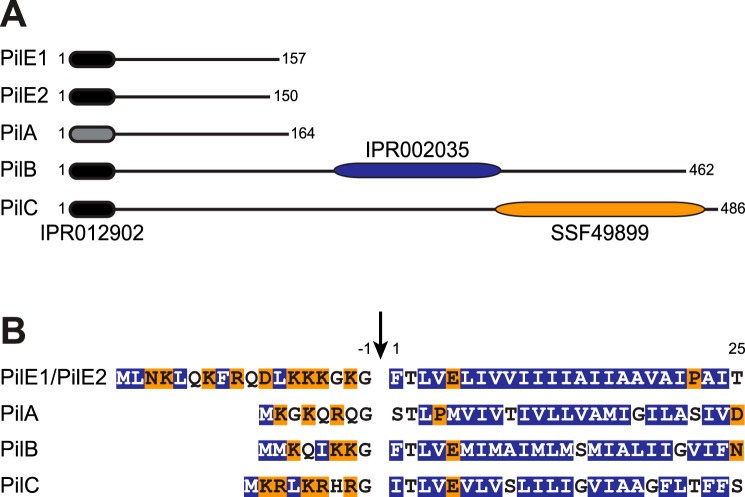
**Bioinformatic analysis of the two major pilins (PilE1 and PilE2) and three pilin-like proteins (PilA, PilB, and PilC) encoded by the *pil* locus in *S. sanguinis* 2908.** *A,* protein architecture of the Pil proteins harboring the N-terminal IPR012902 motif that is part of the class III signal peptide defining type IV pilins (*black rounded rectangle*). In PilA, this motif could be detected only by visual inspection and is therefore represented by a *gray rounded rectangle*. PilB and PilC contain additional C-terminal domains (*blue* and *orange rounded rectangles*). PilB contains a von Willebrand factor type A motif (IPR002035), whereas PilC contains a concanavalin A-like lectin/glucanase structural domain (SSF49899). Proteins have been drawn to scale and the *subscript numbers* indicate their lengths. *B,* N-terminal sequence alignment of the putative class III signal peptides of the above five Pil proteins. The 8–18–aa long leader peptides, which contain a majority of hydrophilic (shaded in *orange*) or neutral (*no shading*) residues, end with a conserved Gly^−1^. Processing by PilD (indicated by the *vertical arrow*) is expected to occur after Gly^−1^. The mature proteins start with a tract of 21 predominantly hydrophobic residues (shaded in *blue*), which forms an extended α-helix that is the main assembly interface within filaments.

All known integral components of Tfp and/or Tff share an N-terminal sequence motif named class III signal peptide (1, 3). It consists of a leader peptide, composed predominantly of hydrophilic amino acids (aa) ending with a conserved Gly^−1^, followed by a stretch of 21 predominantly hydrophobic aa (except for a negatively charged Glu^5^) forming the protruding α1N helix that is the main assembly interface for subunits within filaments (1, 3). Processing by the prepilin peptidase PilD occurs after Gly^−1^. In most Tfp and/or Tff, there are multiple pilin and/or pilin-like proteins (1, 3). Bioinformatic analysis of the proteins encoded by the *pil* locus in *S. sanguinis*, which contains all the genes involved in Tfp biology (24), predicts five pilins and/or pilin-like proteins (PilA, PilB, PilC, PilE1, and PilE2) (Fig. S1*A*). Four proteins (PilB, PilC, PilE1, and PilE2) display a canonical N-terminal IPR012902 motif (26), which is part of the class III signal peptide (Fig. 1*A*). Visual inspection of the sequences of the remaining proteins suggests that PilA has a degenerate class III signal peptide (Fig. 1*B*), which is not identified by the bioinformatic tools available, including PilFind, which is dedicated to the identification of type IV pilins (27).

PilA, PilB, and PilC, all of which are essential for piliation (24), exhibit sequence features distinct from PilE1 and PilE2, the major pilin subunits of *S. sanguinis* Tfp (24). All three proteins have much shorter leader peptides than PilE1/PilE2 (Fig. 1*B*). In addition, although mature PilA has a size (17 kDa) similar to previously studied pilins and pilin-like proteins (3), its putative class III signal peptide is unique for several reasons. The first residue of the predicted mature protein is Ser^1^, there is an unusual Pro^4^, and the highly conserved Glu^5^ is missing. On the other hand, whereas PilB and PilC have canonical class III signal peptides (Fig. 1*B*), both are much larger than classical pilin-like proteins, with mature sizes of 50 and 52.7 kDa, respectively (Fig. 1*A*). This is explained by the presence of bulky domains at the C terminus of PilB and PilC. PilB contains a von Willebrand factor type A motif (IPR002035) (26), whereas PilC contains a concanavalin A-like lectin/glucanase structural domain (SSF49899) (28) (Fig. 1*A*).

Taken together, these findings suggest that PilA, PilB, and PilC are pilin-like proteins, which might be cleaved by PilD and polymerized into *S. sanguinis* Tfp, alongside the major pilins PilE1 and PilE2.

### S. sanguinis Tfp are heteropolymers of N-terminally methylated PilE1 and PilE2

**Figure 2.**
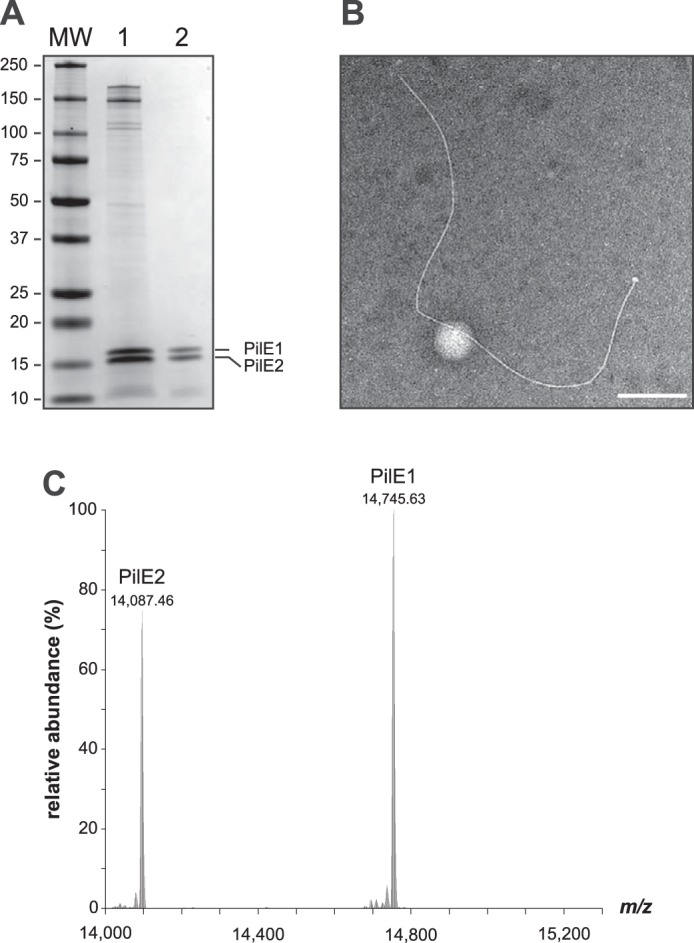
***S. sanguinis* Tfp exhibit a characteristic Tfp morphology and are composed primarily of N terminally methylated PilE1 and PilE2 subunits.** *A,* SDS-PAGE/Coomassie analysis of *S. sanguinis* Tfp purified using previously described (*lane 1*) and enhanced (*lane 2*) purification procedures. Samples were prepared from cultures at similar OD_600_, separated by SDS-PAGE and stained with Coomassie Blue. Identical volumes were loaded in each lane. *MW* indicates the molecular mass marker lane, with masses in kDa. *B*, filament morphology in WT pilus preparations prepared using the enhanced purification procedure as assessed by TEM after negative staining. *Scale bar* represents 200 nm. *C,* top-down mass profiling of purified Tfp from *S. sanguinis*. Shown are deconvoluted molecular mass spectra over the range of 14,000 to 15,300 *m*/*z*. Masses are presented as monoisotopic [M + H]^+^.

As shown previously, purified *S. sanguinis* Tfp consist predominantly of comparable amounts of two proteins, the major pilins PilE1 and PilE2 (24), which share extensive sequence identity (Fig. S2). Tfp, sheared by vortexing, were purified by removing cells/cellular debris by centrifugation before pelleting filaments by ultracentrifugation (24). As assessed by Coomassie staining after SDS-PAGE (Fig. 2*A*), these pilus preparations contain contaminants that originate from cells/cellular debris. To determine the precise composition of *S. sanguinis* Tfp, we improved the purity of our pilus preparations. To remove more debris, we performed one additional centrifugation step and passed the sheared filaments through a 0.22-μm syringe filter before ultracentrifugation. As assessed by SDS-PAGE/Coomassie (Fig. 2*A*), filaments prepared using this enhanced purification procedure were significantly purer, with no visible contaminants. Intriguingly, the morphology of the purified filaments also changed. Although they were previously overwhelmingly thick (12 nm) and wavy (24), most filaments purified using this enhanced procedure display a classical Tfp morphology (1, 4) as assessed by transmission EM (TEM) (Fig. 2*B*). Indeed, they are thin (∼6 nm wide), long (several μm), and flexible, but they do not form large bundles.

In other piliated species, major pilins frequently undergo post-translational modifications (PTM) (3). Their N-terminal residue upon processing is usually methylated and other PTM sometimes include the addition of a variety of glycans and phosphoforms. Methylation is catalyzed by PilD, which is a bifunctional enzyme in many species (8, 9). A bioinformatic analysis shows that *S. sanguinis* PilD contains the IPR010627 motif catalyzing *N*-methylation (Fig. S1*B*), suggesting that the N-terminal Phe^1^ residue in PilE1 and PilE2 is likely to be methylated. Because other PTM cannot be inferred bioinformatically, we used top-down and bottom-up mass spectrometry (MS) to map the PTM of the two major pilins of *S. sanguinis* 2908. Top-down MS analysis of purified filaments showed the presence of two major proteoforms with a ratio of ∼4:3 (PilE1:PilE2) (Fig. 2*C*), with deconvoluted singly charged monoisotopic masses at *m*/*z* 14,745.63 and 14,087.46 Da. These masses are consistent with the predicted theoretical masses of mature PilE1 (14,731.60 Da [M + H]^+^) and PilE2 (14,073.40 Da [M + H]^+^) with the addition of a single N-terminal methyl group (14.01 Da). Bottom-up LC-MS/MS analysis of the two bands excised separately and digested in-gel identified PilE1 and PilE2 with nearly complete sequence coverage (85 and 86%, respectively). The only peptide that was not detected, despite employing several proteolytic enzymes, was the N terminus (^1^FTLVELIVVIIIIAIIAAVAI^21^) (Fig. S2). These MS results strongly suggest that *S. sanguinis* pili consist mainly of a 4:3 ratio of N terminally methylated PilE1 and PilE2 subunits.

**Figure 3.**
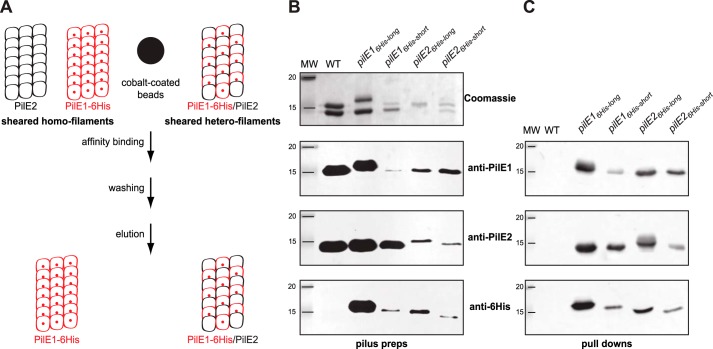
***S. sanguinis* Tfp are heteropolymers composed of PilE1 and PilE2.** *A,* schematics of the affinity-purification strategy. In brief, one of the genes encoding major pilin PilE1 (*red*) or PilE2 (*black*) is engineered to produce a protein fused to an affinity His_6_ tag, *e.g.* PilE1–6His (*small red sphere*). Sheared pili are mixed with cobalt-coated beads (*large black sphere*) and purified by pulldown, which is expected to yield filaments containing both pilins (tagged and untagged) if *S. sanguinis* Tfp are heteropolymers. Conversely, if the filaments are distinct homopolymers, only the tagged filaments will be purified. *B,* analysis of filaments purified by shearing/ultracentrifugation from four unmarked mutants harboring a His_6_ tag fused either to the C terminus of full-length PilE1 and PilE2 (PilE1_6His-long_ and PilE2_6His-long_) or replacing the last seven aa in these pilins (PilE1_6His-short_ and PilE2_6His-short_). The WT strain was included as a control. Samples were prepared from cultures at similar OD_600_, separated by SDS-PAGE and either stained with Coomassie Blue (*upper panel*) or analyzed by immunoblot using anti-PiE1, anti-PilE2, or anti-His_6_ antibodies (*bottom three panels*). *MW* indicates the molecular mass marker lane, in kDa. *C,* immunoblot analysis using anti-PiE1, anti-PilE2, or anti-His_6_ antibodies of sheared filaments that were affinity purified by pulldown. The WT strain was included as a control. Sheared filaments were prepared from cultures adjusted to the same OD_600_, affinity purified, eluted in the same final volume, and identical volumes were loaded in each lane.

Next, we sought to answer the question whether *S. sanguinis* Tfp are heteropolymers of PilE1 and PilE2 or whether two homopolymers co-exist. Recently, using a markerless gene editing strategy (25), we showed that *pilE1* could be engineered *in situ* to encode a protein with a C-terminal His_6_ tag, without affecting piliation or Tfp functionality. We therefore used this property to design an affinity co-purification procedure to answer the above question (Fig. 3*A*). In brief, the intention was to (i) engineer *pilE1* and *pilE2* mutants encoding C terminally His_6_-tagged proteins, (ii) shear the filaments, (iii) affinity-purify (pulldown) sheared filaments containing His_6_-tagged subunits, and (iv) assess whether the untagged pilin co-purifies, suggesting that the filaments are heteropolymers, or does not co-purify, suggesting that two distinct homopolymers co-exist (Fig. 3*A*). We therefore engineered four different variants by either fusing a His_6_ tag to the C terminus of full-length PilE1 and PilE2 (PilE1_6His-long_ and PilE2_6His-long_) or by replacing the last seven aa in these pilins by the tag (PilE1_6His-short_ and PilE2_6His-short_). We first confirmed by SDS-PAGE/Coomassie analysis of purified pilus preparations that the four variants were piliated (Fig. 3*B*), although at various levels. Purified pili contained both tagged and untagged pilins as assessed by immunoblotting using antibodies specific for PilE1 and PilE2 (24), or an anti-His_6_ tag commercial antibody (Fig. 3*B*). When affinity-purified sheared filaments were analyzed by immunoblotting using anti-PilE1, anti-PilE2, or anti-His_6_ antibodies, we found that in each of the four mutants the untagged pilin co-purifies with the tagged pilin (Fig. 3*C*). Importantly, wildtype (WT) untagged filaments cannot be affinity-purified using this procedure, indicating that the pulldown is a His_6_ tag-specific process (Fig. 3*C*). Considering that *S. sanguinis* Tfp show little if any bundling, these findings strongly suggest that they are heteropolymers of PilE1 and PilE2. Taken together, these findings show that *S. sanguinis* Tfp are heteropolymers composed of a 4:3 ratio of PilE1 and PilE2, both harboring a single PTM, *i.e.* a methylated N terminus.

### Major pilin processing and/or assembly into filaments in Gram-positive Tfp follow widely conserved principles

**Figure 4.**
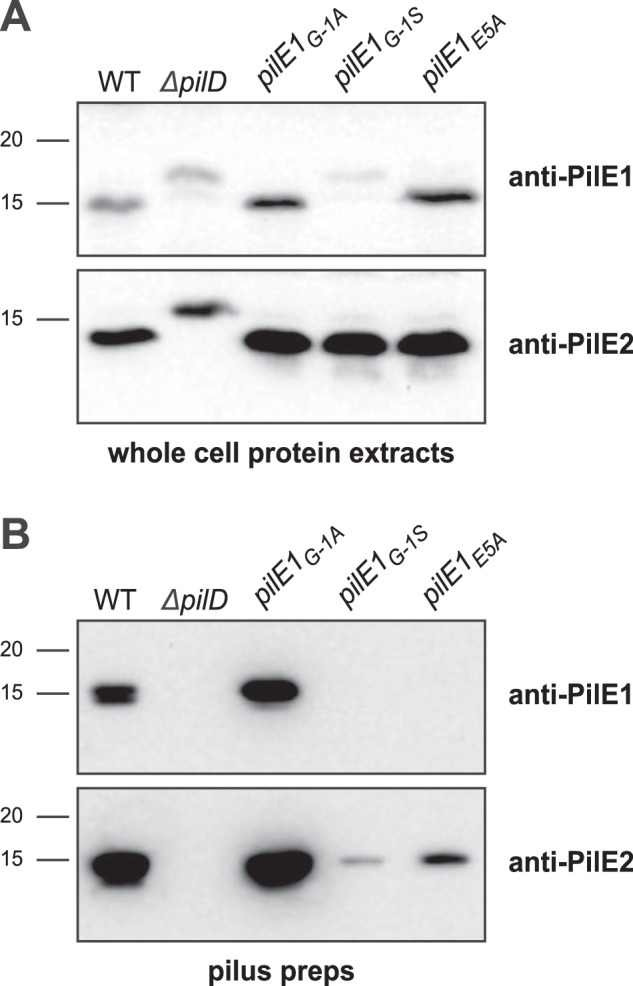
**Processing of *S. sanguinis* major pilin PilE1 by PilD and its assembly in filaments follow widely conserved general principles.** *A,* immunoblot analysis of PilE1 expression and processing by PilD in strains expressing PilE1_G−1A_, PilE1_G−1S_, and PilE1_E5A_ mutants. The WT strain and Δ*pilD* mutant have been included as controls. Whole cell protein extracts were separated by SDS-PAGE and probed using anti-PilE1 antibody or anti-PilE2 antibody as a control. Protein extracts were quantified, equalized, and identical volumes were loaded in each lane. Molecular masses are indicated in kDa. *B,* immunoblot analysis of PilE1 assembly in filaments in strains expressing PilE1_G−1A_, PilE1_G−1S_, and PilE1_E5A_ mutants. The WT strain and Δ*pilD* mutant have been included as controls. Purified Tfp were separated by SDS-PAGE and probed using anti-PilE1 antibody or anti-PilE2 antibody as a control. Samples were prepared from cultures adjusted to the same OD_600_, and identical volumes were loaded in each lane but to improve detection of faint bands, we loaded 20-fold dilutions of WT and PilE1_G−1A_ samples.

Mutagenesis studies of major pilins in several Tfp and/or Tff have identified residues in class III signal peptides key for processing by PilD and/or assembly into filaments (9, 29–31). A common rule has emerged concerning two highly conserved residues Gly^−1^ and Glu^5^ (1). Gly^−1^ is crucial for processing by PilD, whereas Glu^5^ is dispensable for processing but critical for filament assembly (14, 15). Because the importance of these residues has not been assessed for Gram-positive Tfp, we tested it in *S. sanguinis*. Because PilE1 and PilE2 have identical N termini (Fig. 1*B* and Fig. S2), we focused our efforts on PilE1 and used our gene editing strategy (25) to construct markerless mutant strains expressing PilE1 variants in which the Gly^−1^ and Glu^5^ residues would be mutated. Next, we tested whether these mutants proteins were processed by PilD and assembled into filaments. Pilin processing was assessed by immunoblotting in whole-cell protein extracts using the anti-PilE1 antibody (24), *i.e.* processed PilE1 is 14.7 kDa, whereas the unprocessed protein is 16.9 kDa. Assembly into Tfp was assessed by immunoblotting on purified filaments. Originally, we replaced Gly^−1^ by an Ala (PilE1_G−1A_), which had no effect on processing (Fig. 4*A*) or assembly into filaments (Fig. 4*B*). A similar finding was reported in *P. aeruginosa*, and was attributed to the small size of Ala because substitutions with bulkier residues abolished pilin processing (9). Accordingly, when Gly^−1^ was replaced by a Ser (PilE1_G−1S_), PilE1 could not be processed (Fig. 4*A*) or polymerized into filaments, which consisted only of PilE2 (Fig. 4*B*). The marked decrease in PilE2 in pilus preparation suggests a dominant-negative effect of unprocessed PilE1_G−1S_ on PilE2 filament formation. As for the Glu^5^ residue, when it was replaced by an Ala (PilE1_E5A_), PilE1 processing was unaffected (Fig. 4*A*) but the protein could not be polymerized into filaments, which again consisted only of PilE2 (Fig. 4*B*). As above, there was a marked decrease in PilE2 in pilus preparation suggesting a dominant-negative effect of PilE1_E5A_ on PilE2 filament formation. These findings confirm that the widely conserved principles defining how major pilins are processed and assembled into filaments (1) apply to Gram-positive Tfp as well. This further highlights the suitability of *S. sanguinis* as a Gram-positive model to study Tfp biology.

### 3D structure of PilE1 reveals a pilin-fold with an uncommon highly flexible C terminus

**Figure 5.**
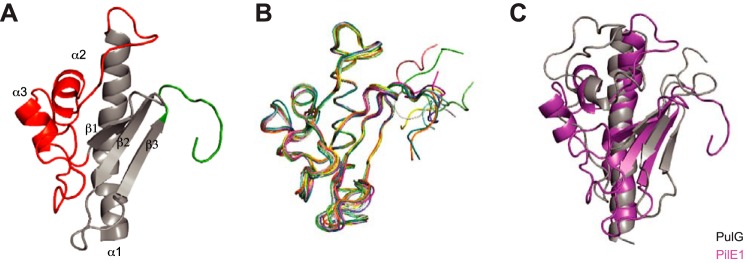
**High-resolution 3D structure of PilE1 reveals a canonical type IV pilin-fold with an uncommon highly flexible C terminus, which more closely resembles pseudopilins.** *A,* cartoon representation of the NMR structure of the globular domain of PilE1. The conserved core in type IV pilins (the N-terminal α-helix and 3-stranded antiparallel β-meander) is depicted in *gray*. Distinctive/key structural features flanking the β-meander are highlighted in color, the α1β1-loop in *red*, and the unstructured C terminus in *green. B,* ribbon representation of the superposition of the ensemble of 10 most favorable PilE1 structures determined by NMR. *C,* superimposed cartoon representations of the globular domains of PilE1 (*magenta*) and *K. oxytoca* pseudopilin PulG (*gray*). The two structures superpose with a root mean square deviation of 5.75 Å over their entire length.

Next, to improve our structural understanding of *S. sanguinis* Tfp, we solved the 3D structure of its major pilins. To facilitate purification, we expressed in *Escherichia coli* the soluble portions of PilE1 (112 aa) and PilE2 (105 aa) fused to an N-terminal His_6_ tag (Fig. S2). This truncated the first 27 residues of the mature proteins, which form the protruding hydrophobic N-terminal α-helix in type IV pilins (1, 3). This procedure allowed us to purify well-folded and soluble proteins using a combination of affinity and gel-filtration chromatography. Because PilE2 shares 78% sequence identity with PilE1 (Fig. S2), we decided to focus on the longest PilE1 and determine its structure by NMR. We isotopically labeled His_6_-PilE1 with ^13^C and ^15^N for backbone and side chain NMR resonance assignment (Table S1) and obtained a high-resolution structure in solution. This structure (Fig. 5*A*) revealed that PilE1 adopts the classical type IV pilin-fold (1, 3). It exhibits a long N-terminal α-helix packed against a β-meander consisting of three anti-parallel β-strands, flanked by distinctive “edges” (Fig. 5*A*), which usually differ between pilins (1, 3). Although the loop connecting α1 and β1 is unexceptional except perhaps for its length (50 aa), the C terminus of the protein (after β3) is striking. Unlike in other pilins in which this region is stabilized by being “stapled” to the last β-strand either by a disulfide bond, a network of hydrogen bonds, or a calcium-binding site (1, 3), the 10-aa long C terminus in PilE1 is unstructured and highly flexible. Although the structures within the NMR ensemble superpose well up to the last β strand (β3), their C termini exhibit different conformations and orientations (Fig. 5*B*). Intriguingly, when compared with structures in the Protein Data Bank, PilE1 was found to be most similar to pseudopilins, which form short Tff, rather than to *bona fide* Tfp subunits. As can be seen in Fig. 5*C*, the structures of PilE1 and PulG, the major pseudopilin in *Klebsiella oxytoca* type II secretion system (T2SS) (32), show extended similarity.

**Figure 6.**
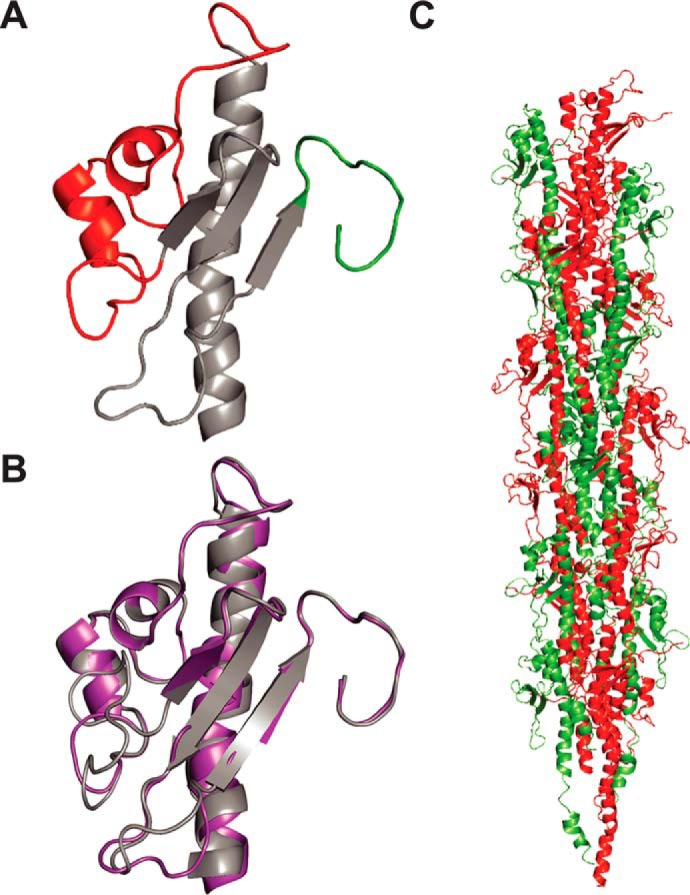
**Modeling shows that full-length PilE1/PilE2 fit readily into available Tfp structures.** *A,* cartoon representation of the homology structural model of PilE2 based on PilE1 structure. The same color codes have been used as described in the legend to Fig. 5*A. B,* superposition of globular domains of PilE1 and PilE2 (*right*) with a root mean square deviation of 0.61 Å. *C,* packing of full-length models of PilE1 and PilE2 into the recently determined structure of gonoccoccal Tfp. Half of the gonoccoccal subunits in the structure were replaced by PilE1 and the other half with PilE2.

Because of their high sequence identity (Fig. S2), we used our PilE1 structure as a template to produce a homology model of PilE2 (Fig. 6*A*). As expected, PilE2 was found to be virtually identical to PilE1 except for its shorter α1β1 loop (Fig. 6*B*), which is explained by the fact that eight residues at the C terminus of this loop in PilE1 are absent in PilE2 (Fig. S2). Finally, after producing full-length models of PilE1 and PilE2 using the full-length gonococcal pilin (33, 34) as a template, we were able to model packing of these pilins within recently determined structures of Tfpa (14, 15) (Fig. 6*C*). This revealed that PilE1 and PilE2 fit readily into these Tfp, which have a similar morphology to *S. sanguinis* filaments. This finding supports the notion that polymerization of pilins into filaments in Gram-positive species also occurs via hydrophobic packing of their α1N helix within the filament core.

Together, these findings show that *S. sanguinis* Tfp obey structural principles common to this class of filaments with some intriguing peculiarities. Although *S. sanguinis* major pilins adopt a canonical type IV pilin-fold, their C terminus appears to be highly flexible, which is uncommon. Moreover, whereas *S. sanguinis* major pilins fit readily within available Tfp structures, they are more similar structurally to pseudopilins than to *bona fide* Tfp-forming pilins.

### PilA, PilB, and PilC are minor pilin components of S. sanguinis Tfp

**Figure 7.**
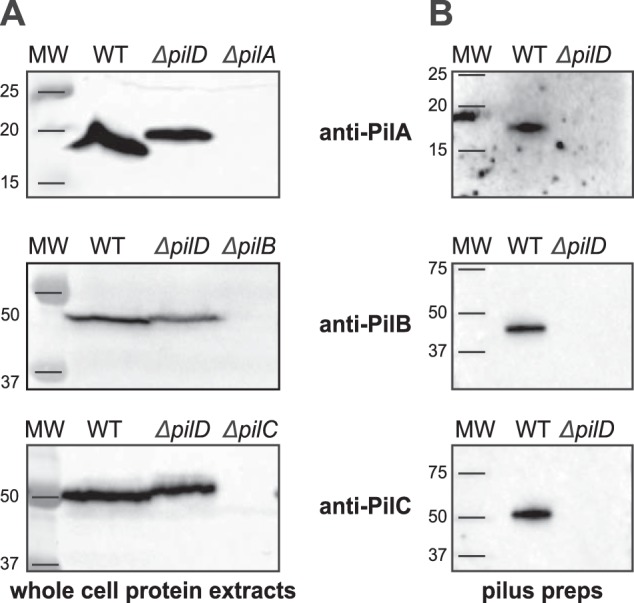
**Pilin-like proteins PilA, PilB, and PilC are processed by PilD and co-purify with Tfp.** *A,* immunoblot analysis of PiA, PilB, and PilC expression and processing by PilD. Whole-cell protein extracts were probed using specific antibodies, which were generated for this study. Protein extracts were quantified, equalized, and equivalent amounts of total proteins were loaded in each lane. Molecular masses are indicated in kDa. *B,* immunoblot analysis of pilus preparations using anti-PiA, anti-PilB, and anti-PilC antibodies. Samples were prepared from cultures adjusted to the same OD_600_ and identical volumes were loaded in each lane.

In all Tff systems, there are in addition to the major pilins several proteins with class III signal peptides, whose role and localization are often unclear (3). To start with experimental characterization of PilA, PilB, and PilC in *S. sanguinis*, we purified each protein and generated antisera. Immunoblotting using whole-cell protein extracts confirmed that the three proteins are expressed because they could be detected in WT, but not in the corresponding deletion mutants (Fig. 7*A*). We then tested whether PilA, PilB, and PilC were processed by PilD, which for PilA was uncertain considering its degenerate class III signal peptide (Fig. 1*B*). Processing by PilD, which removes the leader peptide, is expected to generate mature proteins of 17, 50.5, and 52.8 kDa for PilA, PilB, and PilC, respectively, shorter than their 18-, 51.5-, and 53.9-kDa precursors. Immunoblots confirmed that all three proteins are cleaved by PilD as indicated by the detection of proteins with masses consistent with unprocessed precursors in a Δ*pilD* mutant (Fig. 7*A*). Next, we took advantage of our ability to prepare highly pure Tfp, to determine whether PilA, PilB, and PilC could be detected in pilus preparations by immunoblotting. As can be seen in Fig. 2*A*, although only PilE1 and PilE2 could be detected by SDS-PAGE/Coomassie analysis, PilA, PilB, and PilC are readily detected by immunoblotting in these preparations (Fig. 7*B*). Co-purification of PilA, PilB, and PilC with the filaments depended upon their processing by PilD because these proteins were not detected in sheared fractions prepared from a nonpiliated Δ*pilD* mutant (Fig. 7*B*). In conclusion, findings that PilA, PilB, and PilC are cleaved by PilD and co-purify with Tfp suggest that these three proteins are minor (low abundance) pilin components of *S. sanguinis* Tfp, likely assembled into filaments in a similar fashion to the major subunits PilE1 and PilE2.

### Homopolymeric filaments composed only of PilE1 or PilE2 are able to promote motility, but at different speeds

**Figure 8.**
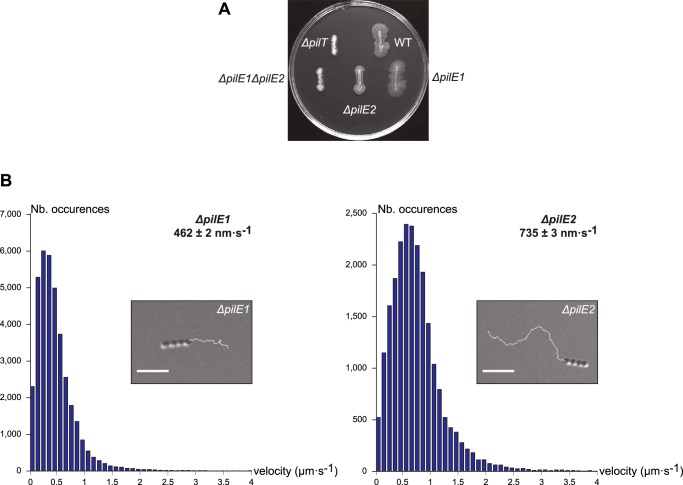
**Δ*pilE1* and Δ*pilE2* mutants produce Tfp capable of powering motility, albeit at different speeds.** *A,* macroscopic motility assay. Spreading zones, or lack thereof, around single and double Δ*pilE1* and Δ*pilE2* mutants. The WT strain and Δ*pilT* mutant have been included as positive and negative controls, respectively. *B,* microscopic motility assay. The histograms represents the distribution curve of velocities (in 100 nm s^−1^ intervals) measured for Δ*pilE1* and Δ*pilE2* mutants. *Insets* are representative 30-s trajectories of movement of small chains of cells. *Scale bar* represents 5 μm. Corresponding movies are available as Supplemental Information (Movies S1 and S2).

As previously reported, whereas a double Δ*pilE1*Δ*pilE2* mutant is nonpiliated, single Δ*pilE1* and Δ*pilE2* mutants produce WT-like filaments consisting of the remaining pilin (24). We therefore wondered whether these homopolymeric filaments are functional and, if so, whether the structural differences between the two pilins (see Fig. 6*B*) would have a functional impact. We compared the ability of the homopolymeric filaments produced by single Δ*pilE1* and Δ*pilE2* mutants to mediate twitching motility. We first found that these mutants still exhibited spreading zones around bacteria grown on agar plates (Fig. 8*A*). This confirms that Tfp consisting exclusively of PilE1 or PilE2 are functional. Motility was next assessed quantitatively at a cellular level by tracking under the microscope the movement of small chains of cells (Fig. 8*B*). As previously reported for the WT (24), both mutants showed “train-like” directional motion mainly parallel to the long axis of bacterial chains. Short duration movies illustrating the movement of Δ*pilE1* and Δ*pilE2* are included as Supplemental Information (Movies S1 and S2). Measurement of instantaneous velocities revealed that, whereas the WT moved at 694 ± 4 nm s^−1^ (mean ± S.E., *n* = 22,957) consistent with previous measurements (24), the mutants moved at 462 ± 2 nm s^−1^ (*n* = 37,001) for Δ*pilE1*, and 735 ± 3 nm s^−1^ (*n* = 22,231) for Δ*pilE2* (Fig. 8*B*). These statistically significant differences suggest that the two pilins are not functionally equivalent with respect to twitching motility.

## Discussion

Their ubiquity in prokaryotes (1) makes Tff an important research topic. A better understanding of the molecular mechanisms governing Tff biology might have practical implications for human health and nanotechnology. Perhaps one of the reasons for our limited understanding of Tff biology is that, historically, these filamentous nanomachines have been studied in just a few Gram-negative bacterial species, all belonging to the same phylum (Proteobacteria) (4). The study of Tff in phylogenetically distant species has the potential to move the field forward, which has recently sparked studies in Archaea (2) and in distant phyla of Bacteria (21). One of the most promising new Tfp models that has emerged is the Gram-positive opportunistic pathogen *S. sanguinis* (24, 25). A recent systematic genetic analysis of Tfp biology in this species, the first to be realized in a non-Proteobacterium, showed that *S. sanguinis* uses a simpler machinery (with fewer components) to assemble canonical Tfpa that generates high tensile forces and power twitching motility (24). In this report, we performed in depth biochemical and structural analysis of *S. sanguinis* filaments, which led to the notable findings discussed below.

The first important achievement in this study is the establishment of one of the most complete biochemical pictures of a Tfp. Critically, this confirms the observed trend (24) that *S. sanguinis* Tfpa are simpler filaments, with fewer components. Indeed, in Gram-negative Tfpa models, in addition to the major pilin there are 7–8 proteins possessing class III signal peptides (4). For example, in *Neisseria meningitidis*, there are four conserved pilin-like proteins required for piliation (PilH, PilI, PilJ, and PilK) whose role (priming filament assembly) (35) and localization (at the tip of the pili or distributed throughout the filaments) (36, 37) remain uncertain, and three species-specific minor pilins (ComP, PilV, and PilX) that are dispensable for piliation but modulate Tfp-associated functions (38–40). In contrast, in *S. sanguinis*, besides PilE1 and PilE2 there are only three Pil proteins possessing class III signal peptides (PilA, PilB, and PilC). As shown here, these proteins are efficiently recognized and post-translationally modified by PilD, which processes their N-terminal leader peptides and (most likely) methylates the first residue of the resulting mature proteins, as demonstrated for PilE1 and PilE2. Processing follows widely conserved principles (1). No other PTM, which frequently decorate major pilins in Gram-negative Tfp are found on *S. sanguinis* PilE1 and PilE2. Importantly, the use of highly pure pilus preparations clearly showed that these five proteins are Tfp subunits. PilE1 and PilE2 are the two major pilins, whereas PilA, PilB, and PilC are three minor pilins. Although the arrangement of the major pilins in the filaments (geometric or stochastic) remains to be determined, this study strongly suggests that *S. sanguinis* Tfp are heteropolymeric structures containing comparable amounts of PilE1 and PilE2, a property not previously reported for Tff. However, the reason for this peculiarity remains unclear because the homopolymeric Tfp assembled by Δ*pilE1* and Δ*pilE2* mutants power efficient twitching motility. A possible explanation might be that PilE1 and PilE2 are important for optimal stability of *S. sanguinis* Tfp as Δ*pilE1* and Δ*pilE2* mutants produce less filaments than WT. On the other hand, what could be the role of the minor subunits of *S. sanguinis* Tfp (PilA, PilB, and PilC)? Although they are required for piliation (24), they are unlikely to prime filament assembly because they are unrelated to the four conserved pilin-like proteins carrying out this process in Gram-negative Tfp (35). Rather, PilA, PilB, and PilC are likely to contribute to filament stability and to modulate Tfp-associated functions. This notion is supported by the unusual presence of large C-terminal domains in PilB and PilC. Interestingly, both the von Willebrand factor type A motif (found in PilB) and the concanavalin A-like lectin/glucanase structural domain (found in PilC) are often involved in binding protein or carbohydrate ligands, suggesting that PilB and PilC are involved in host adhesion in *S. sanguinis*, a property frequently associated with Tfp in other species (4).

The structural information generated on *S. sanguinis* Tfp is the second notable achievement in this study. Our new pilus purification strategy shows that the morphological features of *S. sanguinis* filaments are canonical of Tfpa (4). It is possible that the thick and irregular filaments purified previously (24) were damaged during ultracentrifugation by the presence of cells/cellular debris. Our high-resolution NMR structure of the globular domain of PilE1 confirms that Gram-positive Tfp major subunits adopt the classical type IV pilin-fold (1, 3). The 3D structure of PilE2 is likely to be virtually identical, because of its ∼80% sequence identity to PilE1. Full-length PilE1/PilE2 are therefore expected to adopt the canonical “lollipop” structure (33, 34), because the missing hydrophobic α1N portion can reliably be modeled as a protruding α-helix. Their α1-helix is likely to adopt a gentle S-shaped curve like in the gonococcal major pilin (33, 34), because the helix-breaking Pro^22^ is conserved. Importantly, PilE1/PilE2 fit well in recent cryo-electron microscopy (cryo-EM) reconstructions of several Gram-negative Tfpa (14, 15), which despite different helical parameters display similar packing of the α1N helix within the core of the filaments. Because of the conservation of the helix-breaking Pro^22^, it is likely that the α1N helix will be partially melted between Ala^14^ and Pro^22^ as the pilins in the above reconstructions (14, 15), which is thought to provide flexibility and elasticity to the filaments (41). In addition, this would allow the formation of a salt bridge between Glu^5^ and the methylated Phe^1^ (both conserved in PilE1 and PilE2) of the neighboring pilin. Minor subunits PilB and PilC, which have canonical class III signal peptides similar to PilE1/PilE2 are likely to assemble within filaments in a similar fashion. It is unclear, however, whether the α1N helix would be partially melted, because the helix-breaking Pro^22^ is absent in these proteins. Moreover, it remains to be seen how the bulky C-terminal domains in PilB and PilC, which appear to have been “grafted” by evolution onto a pilin moiety, would be exposed on the surface of the filaments. As for PilA, its unique class III signal peptide, makes it difficult to predict how its α1N helix will be packed in the filament core, especially because of the unusual Pro^4^. Critically, our high-resolution structure of PilE1 also challenges two common assumptions in the field (1, 3). First, in contrast to all previously available pilin structures, the C terminus in PilE1 is unstructured and highly flexible. This explains why it is a permissive insertion site and why it even can be deleted and replaced by a His_6_ tag (25), without interfering with the ability of PilE1/PilE2 to be polymerized into filaments. Therefore, the common rule that the C terminus of major pilins must be stabilized to preserve pilin integrity and its ability to be polymerized (3), is not always true. It cannot be excluded at this point that the major pilins of *S. sanguinis* are an exception as the major pilin of another Tfp-expressing Gram-positive, *Clostridium difficile*, apparently needs to stabilize its C terminus (42). Second, a Tfp-forming major pilin, PilE1, is most similar to pseudopilins that form short filaments in alternative Tff, such as PulG from *K. oxytoca* T2SS (32). This indicates that a major pilin 3D structure cannot be used to predict whether the subunit will form pseudopili or *bona fide* Tfp, which perhaps blurs the lines between different Tff and suggests an even closer evolutionary relationship between these filamentous nanomachines.

In conclusion, by providing an unprecedented global view of a Gram-positive Tfp, this study further cements *S. sanguinis* as a model species that is fast closing the gap with historic Gram-negative Tfp models. Together with our recent reports (24, 25) and *S. sanguinis* exquisite genetic tractability, these findings pave the way for future investigations, which will undoubtedly contribute to improve our understanding of a fascinating filamentous nanomachine almost universal in prokaryotes.

## Experimental procedures

### Strains and growth conditions

Strains and plasmids that were used in this study are listed in Table S2. For cloning, we used *E. coli* DH5α. *E. coli* BL21(DE3) was used for protein purification. *E. coli* strains were grown in liquid or solid lysogeny broth (LB) (Difco) containing, when required, 100 μg/ml of spectinomycin or 50 μg/ml of kanamycin (both from Sigma). The WT *S. sanguinis* 2908 strain and deletion mutants were described previously (24, 25). *S. sanguinis* strains were grown on plates containing Todd Hewitt (TH) broth (Difco) and 1% agar (Difco), incubated at 37 °C in anaerobic jars (Oxoid) under anaerobic conditions generated using Anaerogen sachets (Oxoid). Liquid cultures were grown statically under aerobic conditions in THT (*i.e.* TH broth containing 0.05% Tween 80 (Merck)) to limit bacterial clumping. When required, 500 μg/ml of kanamycin (Km) (Sigma) was used for selection. For counterselection, we used 15 mm *p*-Cl-Phe (Sigma) (25).

Chemically competent *E. coli* cells were prepared as described (43). DNA manipulations were done using standard molecular biology techniques (44). All PCR were done using high-fidelity DNA polymerases from Agilent (see Table S3 for a list of primers used in this study). *S. sanguinis* genomic DNA was prepared from overnight liquid cultures using the kit XIT Genomic DNA from Gram-positive bacteria (G-Biosciences). Strain 2908, which is naturally competent, was transformed as described elsewhere (24, 25).

Unmarked *S. sanguinis* mutants in *pilE1* and *pilE2 in situ* used in this study were constructed using a recently described two-step, cloning-independent, gene editing strategy (25). In brief, in the first step, the target gene was cleanly replaced in the WT, by allelic exchange, with a promoterless pheS*aphA-3 double cassette, which confers sensitivity to *p*-Cl-Phe and resistance to kanamycin. To do this, a splicing PCR (sPCR) product fusing the upstream (amplified with primers F1 and R1) and downstream (amplified with F2 and R2) regions flanking the target gene to pheS*aphA-3 (amplified with *pheS*-F and *aph*-R) was directly transformed into the WT, and allelic exchange mutants were selected on Km-containing plates. Allelic exchange was confirmed for a couple of transformants by PCR. In the second step, the pheS*aphA-3 double cassette was cleanly replaced in this primary mutant, by allelic exchange, with an unmarked mutant allele of the target gene (see below). To do this, an sPCR product fusing the mutant allele to its upstream and downstream flanking regions was directly transformed into the primary mutant and allelic exchange mutants were selected on *p*-Cl-Phe-containing plates. Markerless allelic exchange mutants, which are Km^S^, were identified by re-streaking *p*-Cl-Phe^R^ colonies on TH plates with and without Km. The *pilE1* and *pilE2* mutant alleles encoding proteins with a C-terminal His_6_ tag were engineered by sPCR. We made two constructs for each gene: a long construct in which we fused the His_6_ tag to the C terminus of the full-length protein (sPCR with F1/R3 and F3/R2), and a short construct in which we replaced the last seven aa by the tag (sPCR with F1/R4 and F4/R2). To construct the missense mutants in *pilE1*, we used as a template a pCR8/GW/TOPO plasmid in which the WT gene (amplified with F and R) was cloned (Table S2) and the QuikChange site-directed mutagenesis kit (Agilent) (with complementary primers #1 and #2). Then, the sPCR product for transformation in the primary mutant was produced by fusing the mutant allele (amplified with F and R) to flanking regions upstream (amplified with F1 and R5) and downstream (amplified with F5 and R2).

### SDS-PAGE, antisera, and immunoblotting

*S. sanguinis* whole-cell protein extracts were prepared using a FastPrep-24 homogenizer (MP Biomedicals) and quantified as described elsewhere (24). Separation of the proteins by SDS-PAGE, subsequent blotting to Amersham Biosciences Hybond ECL membrane (GE Healthcare), and blocking were carried out using standard molecular biology techniques (44). To detect PilE1 and PilE2, we used previously described primary rabbit antipeptide antibodies (24). Antisera against PilA, PilB, and PilC were produced for this study by Eurogentec, by immunizing rabbits with purified recombinant proteins (see below). These proteins were then used to affinity purify the antibodies. Primary antibodies were used at between 1/2,000 and 1/5,000 dilutions, whereas the secondary antibody, an ECL horseradish peroxidase-linked anti-rabbit antibody (GE Healthcare) was used at 1/10,000 dilution. Amersham Biosciences ECL Prime (GE Healthcare) was used to reveal the blots. To detect His-tagged proteins, we used a horseradish peroxidase-linked anti-His_6_ antibody (Sigma) at 1/10,000 dilution.

### Tfp purification and visualization

*S. sanguinis* 2908 Tfp were purified as described elsewhere with minor modifications (24). Liquid cultures (10 ml), grown overnight in THT, were used the next day to re-inoculate 90 ml of THT and grown statically until the OD_600_ reached 1–1.5, at which point OD were normalized, if needed. Bacteria were pelleted at 4 °C by centrifugation for 10 min at 6,000 × *g* and pellets were re-suspended in 2 ml of pilus buffer (20 mm Tris, pH 7.5, 50 mm NaCl). This suspension was vortexed for 2 min at full speed to shear Tfp. Bacteria were then pelleted as above, and supernatant containing the pili was transferred to a new tube. This centrifugation step was repeated, before the supernatant was passed through a 0.22-μm pore size syringe filter (Millipore) to remove residual cells and cellular debris. Pili were then pelleted by ultracentrifugation as described (24), resuspended in pilus buffer, separated by SDS-PAGE, and gels were stained using Bio-Safe Coomassie (Bio-Rad). Purified filaments were visualized by TEM after negative staining as described elsewhere (24).

Pulldown purification of His_6_-tagged filaments was done as follows. Fifty μl of Dynabeads His tag isolation and pulldown beads (Life Technologies) were aliquoted in an Eppendorf tube, and pelleted by placing the tube on a magnet for 2 min. After decanting the buffer, beads were washed three times using 500 μl of pilus buffer, mixed with 900 μl of sheared filaments (prepared as for the purification above), and incubated for 10 min on a roller at room temperature. Then, the tube was placed on the magnetic holder for 2 min and the supernatant discarded. Beads were subsequently washed three times with 400 μl of pilus buffer, and finally incubated for 10 min with 100 μl of elution buffer (pilus buffer with 500 mm imidazole) on a roller at room temperature. Then, the tube was placed on the magnetic holder for 2 min, the supernatant was collected and analyzed by SDS-PAGE/Coomassie and/or immunoblotting.

### Proteomics and mass spectrometric analysis of purified Tfp

For the bottom-up MS analysis of purified Tfp, we carefully excised PilE1 and PilE2 protein bands from Coomassie-stained gels and generated enzymatically derived peptides employing four separate enzymes. In brief, as previously described (45), gel pieces were destained and digested overnight with 1 μg of trypsin or Lys-C in 50 mm Na_2_HCO_3_, pH 7.8, at 37 °C, 1 μg of chymotrypsin in 100 mm Tris, 10 mm CaCl_2_·2H_2_O, pH 7.8, at 25 °C, or 0.3 μg of Asp-N in 50 mm Tris, 2.5 mm ZnSO_4_·7H_2_O, pH 8.0, at 25 °C. Generated peptides, which were extracted as previously described (45), were vacuum-concentrated, dissolved in loading buffer (2% acetonitrile, 1% TFA), and desalted using ZIP-TIP tips as instructed by the manufacturer (Millipore). The peptides were eluted with 80% acetonitrile, 1% TFA and vacuum-concentrated. Dried peptide samples were dissolved in 10 μl of 2% acetonitrile, 1% formic acid before analysis by reverse phase LC-MS/MS. Redissolved samples (2–5 μl) were injected into a Dionex Ultimate 3000 nano-UHPLC system (Sunnyvale) coupled online to a QExactive mass spectrometer (Thermo Fisher Scientific) equipped with a nano-electrospray ion source. LC separation was achieved with an Acclaim PepMap 100 column (C18, 3 μm beads, 100 Å, 75-μm inner diameter, 50 cm) and a LC-packing trap column (C18, 0.3-mm inner diameter, 300 Å). The flow rate (15 μl/min) was provided by the capillary pump. A flow rate of 300 nl/min was employed by the nano pump, establishing a solvent gradient of solvent B from 3 to 5% in 5 min and from 5 to 55% in 60 min. Solvent A was 0.1% formic acid, 2% acetonitrile, whereas solvent B was 0.1% formic acid, 90% acetonitrile. The mass spectrometer was operated in data-dependent mode to automatically switch between MS and MS/MS acquisition. Survey full scan MS spectra (from *m*/*z* 200 to 2,000) were acquired with the resolution *r* = 70,000 at *m*/*z* 200, with an automated gain control (AGC) target of 10^6^, and ion accumulation time set at 100 ms. The seven most intense ions, depending on signal intensity (intensity threshold 5.6 × 10^3^) were considered for fragmentation using higher-energy collisional induced dissociation (HCD) at *r* = 17,500 and normalized collision energy (NCE) = 30. Maximum ion accumulation time for MS/MS spectra was set at 180 ms. Dynamic exclusion of selected ions for MS/MS were set at 30 s. The isolation window (without offset) was set at *m*/*z* 2. The lock mass option was enabled in MS mode for internal recalibration during the analysis.

To perform a complete top-down MS analysis of the PilE1 and PilE2 proteoforms present in purified *S. sanguinis* Tfp, three separate methods were employed. For the first method, sample preparation and top-down ESI-MS on a LTQ Orbitrap were performed as previously described (46), and intact protein mass spectra were acquired with a resolution of 100,000 at *m*/*z* 400. For the second method, after initial preliminary testing of a gradient of solvent B from 3 to 55% in 10 min, and from 55 to 85% in 12–35 min, the data were acquired on an LTQ Orbitrap operated in positive ionization mode in the data-dependent mode, to automatically switch between MS and MS/MS acquisition. Survey full scan MS spectra (from *m*/*z* 200 to 2,000) were acquired with a resolution *r* = 100,000 at *m*/*z* 400, with AGC target of 10^6^ and ion accumulation time set at 100 ms. The two most intense ions, depending on signal intensity (intensity threshold 5.6 × 10^3^) were considered for fragmentation using HCD at *r* = 17,500 and NCE = 30. The lock mass option was enabled in MS mode for internal recalibration during the analysis. For the third method, a direct injection nano-ESI top-down procedure was employed. Briefly, the Dionex Ultimate 3000 nano-UHPLC system coupled to the LTQ Orbitrap was reconfigured so that after sample loading onto the loop, valve switching allowed the nanopump to inject directly the sample from the loop into the LTQ Orbitrap MS using an isocratic gradient of 10% methanol, 10% formic acid. Data acquisition was done manually from the LTQ Tune Plus (version 2.5.5 sp2) using *r* = 100,000 and NCE = 400.

Data processing and analysis was done as follows. Bottom-up MS data were analyzed using MaxQuant (version 1.5.2) and the Andromeda search engine against an in-house generated *S. sanguinis* whole proteome database, and a database containing common contaminants. Trypsin, chymotrypsin, Lys-C, Asp-N, and no enzyme (no restriction) were selected as enzymes, allowing two missed cleavage sites. We applied a tolerance of 10 ppm for the precursor ion in the first search, 5 ppm in the second, and 0.05 Da for the MS/MS fragments. In addition to methionine oxidation, protein N-terminal methylation was allowed, in a separate search, as a variable modification. The minimum peptide length was set at 4 aa, and the maximum peptide mass at 5.5 kDa. False discovery rate was set at <0.01. Deconvolution of the PilE1 and PilE2 mass envelopes from top-down analysis was done as previously described (47). Deconvoluted protein masses are reported as monopronated [M + H^+^]. Theoretical masses of PilE1 and PilE2 were determined from available sequences.

### Twitching motility assays

Twitching motility was assessed macroscopically on agar plates as described elsewhere (24). Briefly, bacteria were re-streaked as straight lines on freshly poured TH plates containing 1% Eiken agar (Eiken Chemicals), which were incubated up to several days under anaerobic conditions in a jar, in the presence of water to ensure high humidity. Motility was analyzed microscopically as described elsewhere (24). In brief, bacteria resuspended in THT were added into an open experimental chamber with a glass bottom and grown for 2 h at 37 °C in the presence of 5% CO_2_. The chamber was then transferred to an upright Ti Eclipse microscope (Nikon) with an environment cabinet maintaining the same growth conditions, and movies of the motion of small bacterial chains were obtained and analyzed in ImageJ, as described (24). Cell speed was measured from collected trajectories using Matlab.

### Protein purification

To produce pure PilA, PilB, and PilC proteins for generating antibodies, we cloned the corresponding genes in pET-28b (Novagen) (Table S2). The forward primer was designed to fuse a noncleavable N-terminal His_6_ tag to the soluble portion of these proteins, *i.e.* excluding the leader peptide and the predicted hydrophobic α1N helix. For *pilA*, we amplified the gene from the 2908 genome, whereas for *pilB* and *pilC*, we used synthetic genes (GeneArt), codon-optimized for expression in *E. coli*. Recombinant proteins were purified using a combination of affinity and gel-filtration chromatographies as follows. An overnight liquid culture, in selective LB, from a single colony of *E. coli* BL21 (DE3) transformed with the above expression plasmids, was back-diluted (1:500) the next day in 1 liter of the same medium and grown to an OD_600_ of 0.4–0.6 on an orbital shaker. The temperature was then set to 16 °C, the culture allowed to cool for 30 min, before protein expression was induced overnight by adding 0.1 mm isopropyl 1-thio-β-d-galactopyranoside (Merck Chemicals). The next day, cells were harvested by centrifugation at 8,000 × *g* for 20 min and subjected to one freeze/thaw cycle in binding buffer A (50 mm HEPES, pH 7.4, 200 mm NaCl, 10 mm imidazole, 1× SIGMAFAST EDTA-free protease inhibitor mixture (Sigma)). Cells were disrupted by repeated cycles of sonication, *i.e.* pulses of 5 s on and 5 s off during 3–5 min, until the cell suspension was visibly less viscous. The cell lysate was then centrifuged for 30 min at 17,000 × *g* to remove cell debris. The clarified lysate was then mixed with 2 ml of nickel-nitrilotriacetic acid-agarose resin (Qiagen), pre-washed in binding buffer A, and incubated for 2 h at 4 °C with gentle agitation. This chromatography mixture was then filtered through a Poly-Prep gravity-flow column (Bio-Rad) and washed several times with binding buffer A, before the protein was eluted with elution buffer A (50 mm HEPES, pH 7.4, 200 mm NaCl, 500 mm imidazole, 1× SIGMAFAST EDTA-free protease inhibitor mixture). The affinity-purified proteins were further purified, and simultaneously buffer-exchanged into 50 mm HEPES, pH 7.4, 200 mm NaCl, by gel-filtration chromatography on an Akta Purifier using a Superdex 75 10/300 GL column (GE Healthcare).

For structural characterization of PilE1, we cloned, as above, the portion of *pilE1* encoding the soluble portion of this protein in pET-28b to produce a protein with a noncleavable N-terminal His_6_ tag (Table S2). An overnight pre-culture in LB was back-diluted 1:50 into 10 ml of selective M9 minimal medium, supplemented with a mixture of vitamins and trace elements. This was grown to saturation overnight at 30 °C in an orbital shaker, then back-diluted 1:500 into 1 liter of the same medium containing d-[^13^C]glucose and [^15^N]NH_4_Cl for isotopic labeling. Cells were grown in an orbital shaker at 30 °C until the OD_600_ reached 0.8, then 0.4 mm isopropyl 1-thio-β-d-galactopyranoside was added to induce protein production overnight at 30 °C. As above, cells were then harvested and disrupted in binding buffer B (50 mm Tris-HCl, pH 8.5, 200 mm NaCl, 10 mm imidazole, 1× SIGMAFAST EDTA-free protease inhibitor mixture). The protein was first purified by affinity chromatography and eluted in 50 mm Tris-HCl, pH 8.5, 200 mm NaCl, 200 mm imidazole, 1× SIGMAFAST EDTA-free protease inhibitor mixture. It was then further purified and buffer-exchanged into 50 mm Na_2_HPO_4_/NaH_2_PO_4_, pH 6, 200 mm NaCl by gel-filtration chromatography using a Superdex 75 10/300 GL column.

### NMR structure determination of PilE1

Structure determination of PilE1 was done by NMR, essentially as described (48). In brief, isotopically labeled purified His_6_-PilE1 was concentrated to ∼750 μm in NMR buffer (50 mm Na_2_HPO_4_/NaH_2_PO_4_, pH 6, 50 mm NaCl, 10% D_2_O). A full set of triple resonance NMR spectra was recorded on a Bruker Avance III 800 MHz spectrometer equipped with triple resonance cryoprobes at 295 K, and processed with NMRPipe (49). Backbone assignments were completed using a combination of HBHA, HNCACB, HNCO, HN(CA)CO, and CBCA(CO)NH experiments using NMRView (One Moon Scientific) (50). Side chain resonance assignments were obtained from a combination of CC(CO)NH, HC(C)H-TOCSY, and (H)CCH-TOCSY experiments using in-house software developed within NMRView (51). Distance restraints were obtained from 3D ^1^H,^1^H,^15^N-NOESY and ^1^H,^1^H,^13^C-NOESY spectra and used for structure calculations in ARIA 2.3 (52), along with dihedral angle restraints obtained from chemical shift values calculated using the TALOS+ server (53). For each round of six calculations, 100 structures were calculated over eight iterations. In the final iteration, the 10 lowest energy structures were submitted to a water refinement stage to form the final structural ensemble.

### Bioinformatics

All the sequences were from the genome of *S. sanguinis* 2908 (24). Protein alignments were done using the Clustal Omega server at EMBL-EBI, with default parameters. Pretty-printing and shading of alignment files was done using the BOXSHADE server at ExPASy. Prediction of functional domains was done by scanning the protein sequence either against InterPro protein signatures (26), or against the SUPERFAMILY database of structural protein domains (28). Both analyses were done using default parameters. MODELLER was used for modeling protein 3D structures (54). In brief, a homology model for full-length PilE1 was produced using MODELLER in multiple template mode, with the N-terminal 25 residues of PilE from the gonococcal Tfp (15) and a representative of the PilE1 NMR ensemble determined in this study. This PilE1 model was then used as a template to produce a PilE2 model using MODELLER in single template mode. These two molecules were then superimposed onto a single chain each of the gonococcal filament cryo-EM reconstruction (15), using COOT (55), and using the SSM superpose function. Sections of the polypeptide were then refined into the electron densities using the real space refine function in COOT to adjust fitting of PilE1/PilE2 monomers into the helical filament. Multiple copies of these models were then used to produce the final representation. Structural homologs of PilE1 were identified by scanning the Protein Data Bank using the Dali server (56).

## Author contributions

J.-L. B., I. G., J. H. A., N. B., and V. P. data curation; J.-L. B., I. G., J. H. A., M. K., N. B., S. M., and V. P. formal analysis; J.-L. B., I. G., J. H. A., I. S., E. H., A. M. J. H., V. J. G., C. R., and V. P. investigation; J.-L. B., I. G., J. H. A., M. K., N. B., S. M., and V. P. methodology; J.-L. B., I. G., J. H. A., E. H., A. M. J. H., V. J. G., C. R., M. K., N. B., S. M., and V. P. writing-review and editing; M. K., N. B., S. M., and V. P. resources; M. K., N. B., S. M., and V. P. project administration; N. B. and V. P. supervision; S. M. and V. P. funding acquisition; V. P. conceptualization; V. P. writing-original draft.

## Supplementary Material

Supporting Information

## Footnotes

4 The abbreviations used are:

Tfp: type IV pili
Tff: type IV filament(s)
PTM: post-translational modification(s)
T2SS: type II secretion system
TH: Todd Hewitt
LB: lysogeny broth
TEM: transmission electron microscopy
cryo-EM: cryo-electron microscopy
sPCR: splicing PCR
AGC: automated gain control
HCD: higher-energy collisional induced dissociation
NCE: normalized collision energy
aa: amino acid(s).
