# Supplementary material for: Global biochemical and structural analysis of the type IV pilus from the Gram-positive bacterium Streptococcus sanguinis
Source: J Biol Chem. 2019 Mar 5;294(17):6796–808. doi: 10.1074/jbc.RA118.006917 (PMC6497953; doi:10.1074/jbc.RA118.006917)
Supplement: Supporting Information [file supp_294_17_6796__index.html]

Global biochemical and structural analysis of the type IV pilus from the Gram-positive bacterium Streptococcus sanguinis — Gram-positive type IV pili — Global biochemical and structural analysis of the type IV pilus from the Gram-positive bacterium Streptococcus sanguinis — Gram-positive type IV pili — Supporting Information 

# Global biochemical and structural analysis of the type IV pilus from the Gram-positive bacterium *Streptococcus sanguinis*

## Supporting Information

- Supporting Information - Supporting Figures and Tables.
- Supporting movie - Movie S1
- Supporting movie - Movie S2
