# Supplementary material for: Global biochemical and structural analysis of the type IV pilus from the Gram-positive bacterium Streptococcus sanguinis
Source: J Biol Chem. 2019 Mar 5;294(17):6796–808. doi: 10.1074/jbc.RA118.006917 (PMC6497953; doi:10.1074/jbc.RA118.006917)
Supplement: Supporting Information [file supp_RA118.006917_142251_2_supp_292987_pnlnny.pdf]

Global biochemical and structural analysis of the type IV pilus from the Gram-positive bacterium *Streptococcus sanguinis*

Jamie-Lee Berry[1], Ishwori Gurung[1], Jan Haug Anonsen[2,3], Ingrid Spielman[4,5], Elliot Harper[1], Alexander M. J. Hall[1], Vivianne J. Goosens[1], Claire Raynaud[1], Michael Koomey[3], Nicolas Biais[4,5], Stephen J. Matthews[6], Vladimir Pelicic[1]

From the [1]Medical Research Council Centre for Molecular Bacteriology and Infection, Imperial College London, London, United Kingdom; [2]Department of Biological Sciences, Proteomics and Mass Spectrometry Unit, University of Oslo, Oslo, Norway; [3]Department of Biological Sciences, Center for Integrative Microbial Evolution, University of Oslo, Norway; [4]Department of Biology, Brooklyn College of the City University of New York, New York, USA; [5]The Graduate Center of the City University of New York, New York, USA; [6]Centre for Structural Biology, Imperial College London, London, United Kingdom

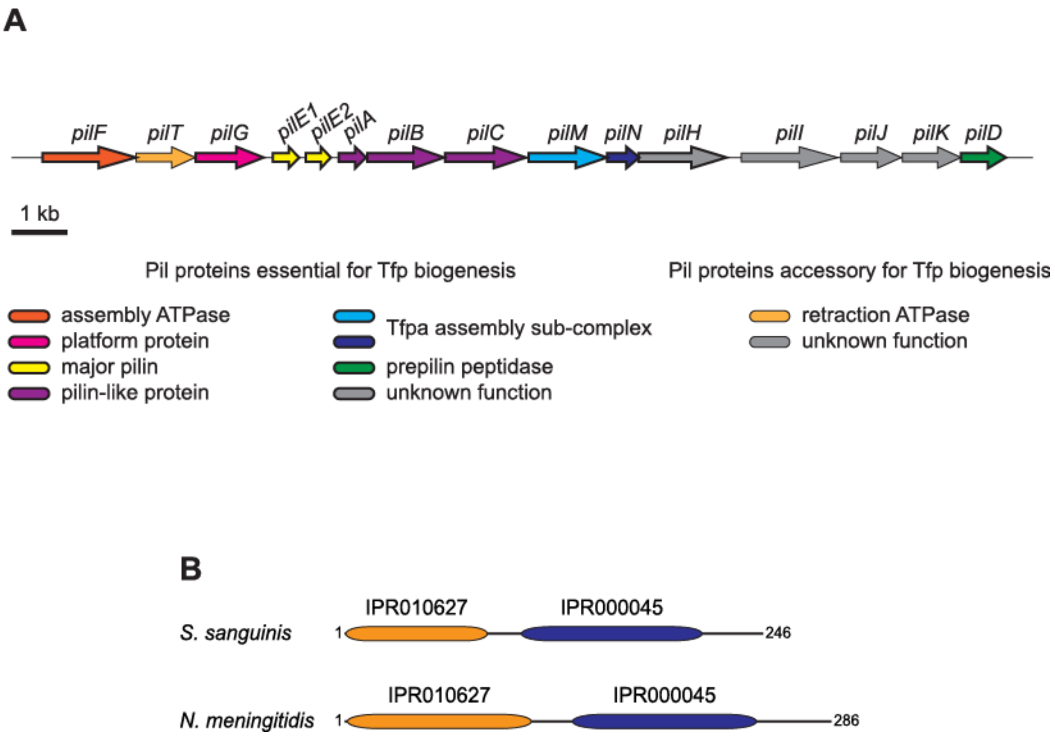

Figure S1. Bioinformatic analysis of the genes in the *pil* locus of *S. sanguinis* 2908 and of its prepilin peptidase PilD. (**A**) Gene organisation in the *pil* locus in *S. sanguinis* 2908. All the genes are drawn to scale and the scale bar represents 1 kb. Genes essential for Tfp biogenesis are boxed by a thick line. (**B**) Protein architecture of the prepilin peptidases in *S. sanguinis* and *N. meningitidis*. The N-terminal IPR010627 motif (orange rounded rectangle) catalyses N-methylation, while the C-terminal IPR000045 motif (blue rounded rectangle) catalyses proteolytic processing of pilins. Proteins have been drawn to scale and the subscript numbers indicate protein length.

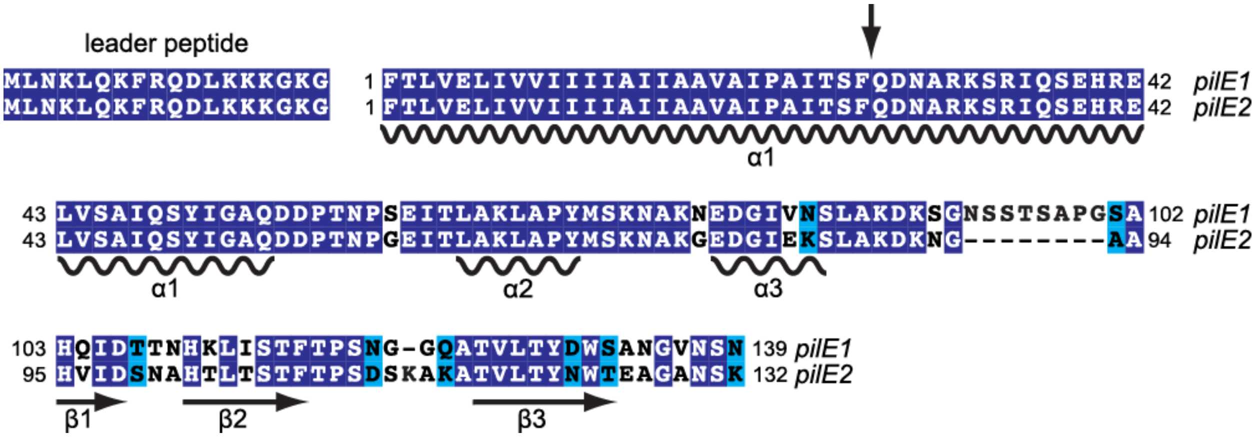

Figure S2. Sequence alignment of the major pilins PilE1 and PilE2 in *S. sanguinis* 2908. Residues are shaded in dark blue (identical), light blue (conserved) or unshaded (different). Structural features - α-helices and β-strands - are indicated below the sequences. The vertical arrow indicates the N-terminal portion of PilE1 and PilE2 that was truncated to facilitate protein purification.

Movies S1. Cellular motility of a *ΔpilE1* mutant. A small chain of cells attached to a coverslip was imaged for 30 sec. The scale bar represents 5 µm.

Movies S2. Cellular motility of a *ΔpilE2* mutant. A small chain of cells attached to a coverslip was imaged for 30 sec. The scale bar represents 5 µm.

Table S1. NMR structural statistics.

**6His-PilE1**	
Number of distance restraints	1,750
intra-residual	712
sequential	416
medium range	287
long range	335
NOE violations >0.5 Å (%)	0.85
Dihedral violations >5º (%)	0
Ramachandran favoured (%)	84.4
Ramachandran allowed (%)	13.5
Ramachandran generously allowed (%)	0.7
Ramachandran disallowed (%)	1.3

Table S2. Strains and plasmids used in this study.

Name	Details	Source
***E. coli* strains**		
DH5α	used for cloning	
BL21(DE3)	used for protein expression and purification	
***S. sanguinis* strains**		
2908	sequenced WT isolate	(1)
ΔpilA	*ΔpilA::aphA-3* deletion mutant	(1)
ΔpilB	*ΔpilB::aphA-3* deletion mutant	(1)
ΔpilC	*ΔpilC::aphA-3* deletion mutant	(1)
ΔpilD	*ΔpilD::aphA-3* deletion mutant	(1)
ΔpilE1	*ΔpilE1::aphA-3* deletion mutant	(1)
ΔpilE1 primary mutant	*ΔpilE1::pheS*aphA-3* deletion mutant	(2)
$pilE1_{G\text{-}1A}$	*pilE1* point mutant expressing $\text{PilE1}_{\text{G-1A}}$	this study
$pilE1_{G\text{-}1S}$	*pilE1* point mutant expressing $\text{PilE1}_{\text{G-1S}}$	this study
$pilE1_{E5A}$	*pilE1* point mutant expressing $\text{PilE1}_{\text{E5A}}$	this study
$pilE1_{6His\text{-}short}$	*pilE1* point mutant expressing 6His-tagged $\text{PilE1}_{\text{short}}$	this study
$pilE1_{6His\text{-}long}$	*pilE1* point mutant expressing 6His-tagged $\text{PilE1}_{\text{long}}$	(2)
ΔpilE2	*ΔpilE2::aphA-3* deletion mutant	(1)
ΔpilE2 primary mutant	*ΔpilE2::pheS*aphA-3* deletion mutant	this study
$pilE2_{6His\text{-}short}$	*pilE2* point mutant expressing 6His-tagged $\text{PilE2}_{\text{short}}$	this study
$pilE2_{6His\text{-}long}$	*pilE2* point mutant expressing 6His-tagged $\text{PilE2}_{\text{long}}$	this study
ΔpilE1ΔpilE2	*ΔpilE1ΔpilE2::aphA-3* double deletion mutant	(1)
ΔpilT	*ΔpilT::aphA-3* deletion mutant	(1)
**Plasmids**		
pCR8/GW/TOPO	TA cloning vector	Invitrogen
TOPO-*pheS*aphA-3*	*pheS*aphA-3* double cassette in pCR8/GW/TOPO	(2)
TOPO-*pilE1*	full-length *pilE1* in pCR8/GW/TOPO	this study
TOPO-$pilE1_{G\text{-}1A}$	$pilE1_{G\text{-}1A}$ in pCR8/GW/TOPO	this study
TOPO-$pilE1_{G\text{-}1S}$	$pilE1_{G\text{-}1S}$ in pCR8/GW/TOPO	this study
TOPO-$pilE1_{E5A}$	$pilE1_{E5A}$ in pCR8/GW/TOPO	this study
pMK-*pilB*	codon-optimised *pilB* in pMK	GeneArt
pMK-RQ-*pilC*	codon-optimised *pilC* in pMK-RQ	GeneArt
pET-28b	T7-based expression vector	Novagen
pET28-*pilA*	pET-28b derivative for expressing 6His-$\text{PilA}_{33\text{-}164}$	this study
pET28-*pilB*	pET-28b derivative for expressing 6His-$\text{PilB}_{37\text{-}462}$	this study
pET28-*pilC*	pET-28b derivative for expressing 6His-$\text{PilC}_{34\text{-}486}$	this study
pET28-*pilE1*	pET-28b derivative for expressing 6His-$\text{PilE1}_{46\text{-}157}$	this study
pET28-*pilE2*	pET-28b derivative for expressing 6His-$\text{PilE2}_{46\text{-}150}$	this study

Table S3. Primers used in this study.

Name	Sequence
**Cloning in pET-28b**	
pilA-pETF	gggccatggatcatcatcatcatcatcatGATACAGGGCAAAGCCAGAC
pilA-pETR	cccgtcgacTTACTTCTGTGCCGATCTCAA
pilB-pETF	gggccatggatcatcatcatcatcatcatAGCAGCCGTGAACTGATTGA
pilB-pETR	cccggatccTTACGGACCGCTAACAAACC
pilC-pETF	gggccatggatcatcatcatcatcatcatAATAACATTCTGCGTCAGCGTAGCCA
pilC-pETR	cccggatccTTAGCTTGCTTTGTATTTATCGC
pilE1-pETF	ggccatggatcatcatcatcatcatcatCAAGATAACGCTCGTAAGAGCC
pilE1-pETR	ccggatccTTAGTTTGAGTTTACACCATTAGCAGA
pilE2-pETF	ggccatggatcatcatcatcatcatcatCAAGATAACGCTCGTAAGAGCC
pilE2-pETR	ccggatccTTATTTTGAATTAGCACCAGCTTCG
**Cloning in pCR8/GW/TOPO**	
pilE1-F	AGGAACAAAACAAATGCCCCT
pilE1-R	TCTCAAATGCAGGGTTTTACTACA
**Site-directed mutagenesis**	
pilE1$_{G\text{-}1A}$#1	GACTTGAAGAAAAAAGGTAAAG**C**TTTTACCTTGGTTGAGTTGATC
pilE1$_{G\text{-}1A}$#2	GATCAACTCAACCAAGGTAAAA**G**CTTTACCTTTTTTCTTCAAGTC
pilE1$_{G\text{-}1S}$#1	GACTTGAAGAAAAAAGGTAAA**A**GTTTTACCTTGGTTGAGTTGATC
pilE1$_{G\text{-}1S}$#2	GATCAACTCAACCAAGGTAAAAC**T**TTTACCTTTTTTCTTCAAGTC
pilE1$_{E5A}$#1	GTAAAGGTTTTACCTTGGTTG**C**GTTGATCGTGGTAATTATC
pilE1$_{E5A}$#2	GATAATTACCACGATCAAC**G**CAACCAAGGTAAAACCTTTAC
**Engineering *S. sanguinis* mutants**	
pheS-F	ATGACGAAAACGATTGAAGAAC
aph-R	CTAAAACAATTCATCCAGTAAAA
pilE1-F1	CAGGCCGGTGAAAAGACTG
pilE1-R1	GTTCTTCAATCGTTTTCGTCATTTTGAATAGATCTCCTGTTTTT
pilE1-F2	TTTTACTGGATGAATTGTTTTAGCGACTGGTCTGCTAATGGTG
pilE1-R2	GCTCTGTTGAAGGATCCACG
pilE1-R3	TTAGTGATGGTGATGGTGATGGTTTGAGTTTACACCATTAGCAG
pilE1-F3	CATCACCATCACCATCACTAATTGTCAAATCATCTAAATAAGATGTA
pilE1-R4	TTAGTGATGGTGATGGTGATGAGACCAGTCGTAGGTCAAAAC
pilE1-F4	CATCACCATCACCATCACTAATTGTCAAATCATCTAAATAAGATGTA
pilE1-R5	AGGGGCATTTGTTTTGTTCCT
pilE1-F5	TGTAGTAAAACCCTGCATTTGAGA
pilE2-F1	GGAACGTCTGACAGGGATGA
pilE2-R1	GTTCTTCAATCGTTTTCGTCATATGTATTTTCTCCTAATGTTTTTATG
pilE2-F2	TTTTACTGGATGAATTGTTTTAGACCGAAGCTGGTGCTAATTC
pilE2-R2	TACCATCCGCAGAAAGACCA
pilE2-R3	TTAGTGATGGTGATGGTGATGTTTTGAATTAGCACCAGCTTC
pilE2-F3	CATCACCATCACCATCACTAATAACTTGAATTAATTTGAGTTATTCAT
pilE2-R4	TTAGTGATGGTGATGGTGATGGGTCCAGTTGTATGTTAAAACG
pilE2-F4	CATCACCATCACCATCACTAATAACTTGAATTAATTTGAGTTATTCAT

Overhangs are in lower case, with restriction sites underlined. Mismatched bases generating point mutations are in bold upper case.

References

1. Gurung, I., Spielman, I., Davies, M. R., Lala, R., Gaustad, P., Biais, N., and Pelicic, V. (2016) Functional analysis of an unusual type IV pilus in the Gram-positive *Streptococcus sanguinis*. *Mol. Microbiol.* **99,** 380-392
2. Gurung, I., Berry, J. L., Hall, A. M. J., and Pelicic, V. (2017) Cloning-independent markerless gene editing in *Streptococcus sanguinis*: novel insights in type IV pilus biology. *Nuc. Acids Res.* **45,** e40
